# Unexplained Dyspnea in a Young Adult with Epstein–Barr Virus Infectious Mononucleosis: Pulmonary Involvement or Co-Infection with *Mycoplasma* *pneumoniae* Pneumonia?

**DOI:** 10.3390/jcm6090083

**Published:** 2017-09-04

**Authors:** Burke A. Cunha, Scarlet Herrarte Fornos

**Affiliations:** 1 Infectious Disease Division, Winthrop-University Hospital Mineola, NY 11501, USA; tusalile@aol.com; 2 State University of New York, School of Medicine, Stony Brook, NY 11790, USA

**Keywords:** bacterial and viral co-infection, non-exudative pharyngitis, community-acquired pneumonia, atypical pneumonia, cold agglutinins, atypical lymphocytosis, elevated serum transaminases

## Abstract

Clinically, in young immunocompetent adults, Epstein-Barr virus (EBV) usually manifests as infectious mononucleosis (IM). Typical clinical findings of EBV IM include fever, profound fatigue, pharyngitis, bilateral posterior cervical adenopathy, and splenomegaly. Respiratory involvement with EBV IM may occur, but is distinctly rare. We present a case of a 20 year old female who with classic EBV IM, but was inexplicably dyspneic and hypoxemic. Further diagnostic testing confirmed co-infection with *Mycoplasma pneumoniae*. As a non-zoonotic atypical community-acquired pneumonia (CAP), *M. pneumoniae* may rarely be accompanied by severe hypoxemia and even acute respiratory distress syndrome. She represented a diagnostic dilemma regarding the cause of her hypoxemia, i.e., due to EBV IM with pulmonary involvement or severe *M. pneumoniae* CAP. The patient slowly recovered with respiratory quinolone therapy.

## 1. Introduction

Community-acquired pneumonia (CAP) is nearly always due to a single respiratory pathogen. In adults, the most common example of co-infection is with influenza A and *S. aureus*. Excluding influenza, true CAP co-infections are rare. Co-infection with Legionnaire’s disease and *S. pneumoniae* is uncommon, but it has been reported that CAP co-infections may involve different types of pathogens, i.e., viral and bacterial, but rarely are co-infections due to the same type of pathogen. In children, and to a lesser extent adults, co-colonization, particularly with respiratory viruses, is common. The demonstration of viruses from respiratory secretions does not, per se, prove a bona fide viral CAP co-infection.

We present a most interesting case representing a diagnostic dilemma regarding the cause of her dyspnea. Her hypoxemia was due to one or other pathogen or were her respiratory findings the result of the combined effects of both disorders?

## 2. Case

A 20-year-old female presented to the Emergency Department with fever, fatigue, sore throat, and shortness of breath ×4 days. She particularly complained of marked dyspnea on exertion (DOE). She was a normal host with no significant past medical history and was a non-smoker. On admission, she was febrile (100.6 °F) with a respiratory rate of 20/min. Her physical exam was unremarkable except for a minimal erythematous pharynx and bilateral posterior cervical adenopathy. Her oxygen saturation (S_a_O_2_) on room air was 81%. 

**Figure 1:**
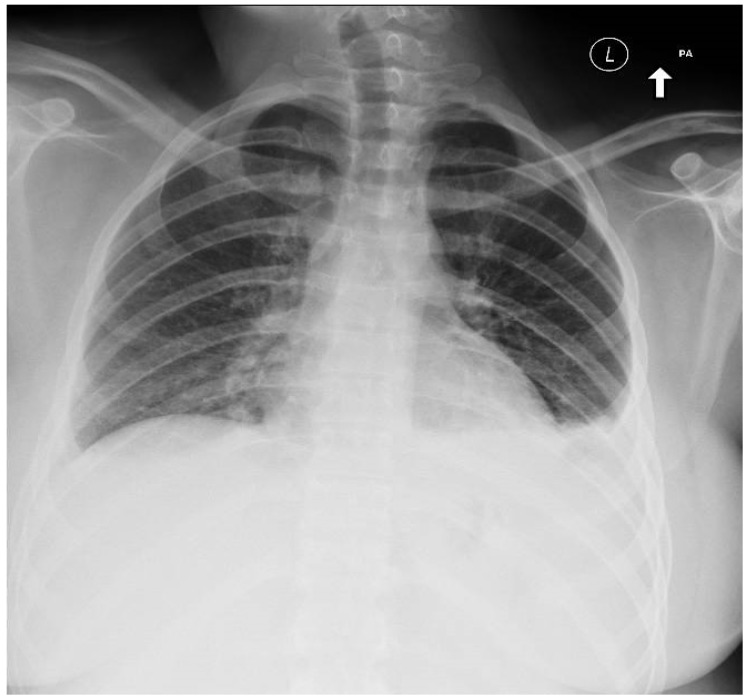
Chest film showing no infiltrates, and no hilar adenopathy with small left sided pleural effusion.

**Figure 2:**
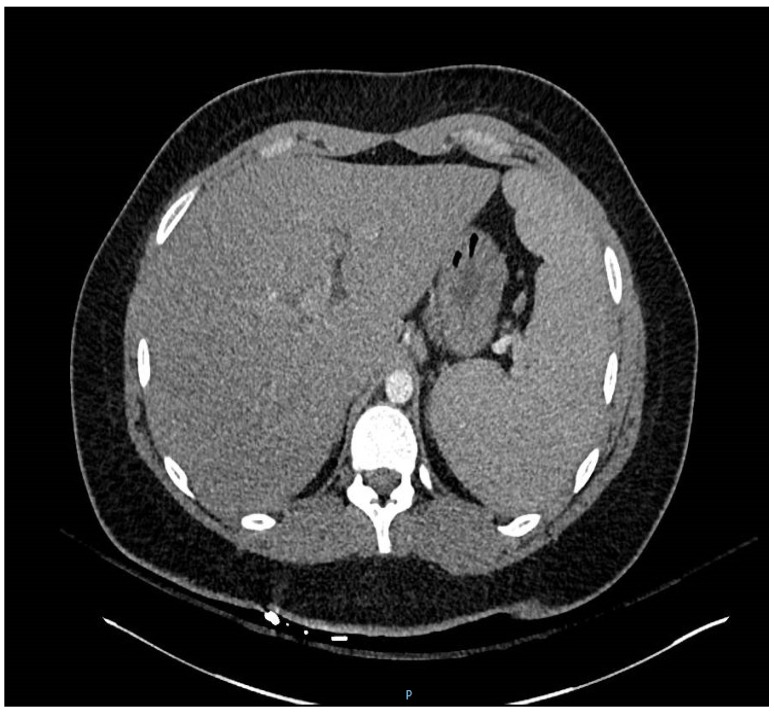
Chest computed tomography (CT) scan showing marked splenomegaly.

Because of fever, sore throat and dyspnea, oropharyngeal swab for respiratory panel PCR Film Array (Genexpert, Paris, France) was done and was negative for influenza, respiratory viruses and *Mycoplasma pneumoniae*. Her complete blood count (CBC) was unremarkable except marked atypical lymphocytosis 21% (*n* < 5%). Routine laboratory tests were also unremarkable except for mildly elevated serum transaminases. The MonoSpot test was positive. Chest X-ray (CXR) showed no infiltrates and a small left pleural effusion (Figure 1). Chest computed tomography (CT) scan revealed multiple bilateral lower-lobe infiltrates and splenomegaly (15.2 cm × 17.1 cm) (Figure 2). Her EBV VCA IgM titer was 72.8 U/mL (*n* ≤ 36 U/mL), and her EBV VCA IgG titer was 402 U/mL (*n* ≤ 18 U/mL). 

**Figure 3:**
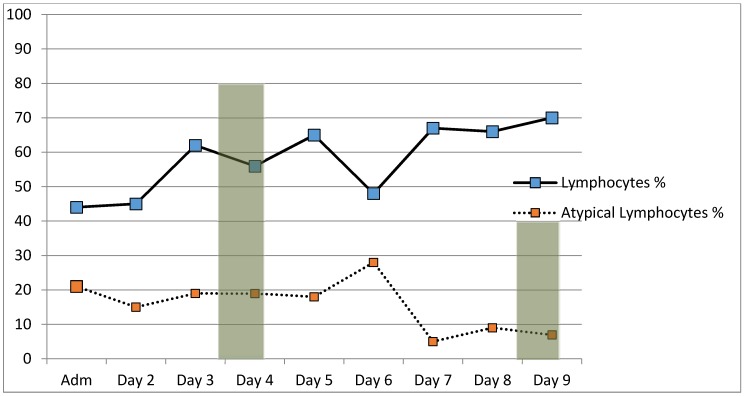
Serial atypical lymphocyte and lymphocyte counts during hospitalization in a patient with Epstein–Barr virus infectious mononucleosis and *M. pneumoniae*.

However, clearly the patient had EBV infectious mononucleosis (IM), but it was difficult to ascribe the patient’s hypoxemia/dyspnea to EBV IM alone. Accordingly, she was empirically treated for a possible “atypical pneumonia” with levofloxacin 500 mg daily × 2 weeks Her *Mycoplasma pneumoniae* IgM titer was elevated at 3.02 (*n* ≤ 0.9) and her IgG titer was 402 (*n* < 18). Her cold agglutinin titer was highly elevated at 1:256 (*n* ≤ 16). *Mycoplasma pneumoniae* titers repeated on hospital day (HD) #9, decreased the IgM titer 2.29 (*n* < 0.9), with a IgG titer of 2.29 (*n* ≤ 1.10). Repeat cold agglutinin titer was 1:128 (Figure 3).

Repeat EBV titers included an EBV VCA IgM titer of 96.8 U/mL (*n* ≤ 36 U/mL), and a EBV viral capsid antigen (VCA) IgG titer of >600 U/mL (*n* ≤ 18 U/mL), Repeat MonoSpot test remained positive. 

## 3. Discussion

This normal host young adult presented with fever, fatigue and sore throat. Clinically, these findings plus bilateral posterior adenopathy pointed to Epstein–Barr virus IM as the likely diagnosis. Her mildly elevated serum transaminases were likely due to her EBV IM since liver involvement with *M. pneumoniae* is rare [1,2,3].

The degree of her atypical lymphocytosis limited the differential diagnosis to EBV or cytomegalovirus (CMV) [1]. While the CXR showed only a small left-sided pleural effusion, chest CT showed bilateral lower-lobe infiltrates without hilar/mediastinal adenopathy and marked splenomegaly. Not unsurprisingly, her MonoSpot test was positive and her EBV VCA IgM titers were highly elevated. The mildly elevated serum transaminases were consistent with diagnosis of EBV IM [1]. 

Given that her hypoxemia with an essentially unremarkable CXR suggested superimposed viral community-acquired pneumonia (CAP), respiratory panel PCR done on an oropharyngeal swab specimen ordered and was negative for influenza, other respiratory viruses as well as *M. pneumoniae.*

In the absence of respiratory pathogen co-infection, we reviewed the literature on the pulmonary manifestations of EBV IM. More common in children, EBV may involve the lung parenchyma resulting in dyspnea [4,5,6]. (Table 1) During her initial work-up for a viral or atypical CAP to explain her pulmonary symptoms, *M. pneumoniae* titers were ordered, along with cold agglutinin titers that were reported later in her hospitalization. Until the elevated *M. pneumoniae* and cold agglutinin titers were reported, it was thought that her hypoxemia was explained by EBV lung involvement [6,7,8]. EBV lung involvement alone may present as severe pneumonia with hypoxemia/dyspnea. The additional findings of highly elevated *M. pneumoniae* IgM titers and cold agglutinin titers were a diagnostic dilemma in this case. Could either EBV IM or *M. pneumoniae* alone explain her dyspnea or was this a co-infection?

A minimally elevated *M. pneumoniae* titer may be difficult to interpret, but a highly elevated *M. pneumoniae* IgM is diagnostic particularly when accompanied by highly elevated cold agglutinin titers (>1:64). While some respiratory viruses may elaborate cold agglutinins, titers are usually low, e.g., 1:4–1:16, but not >1:64 [2,3] (Table 2). Therefore, although the diagnosis of *M. pneumoniae* CAP was firm, the diagnostic dilemma remained, i.e., which process was primarily responsible for her hypoxemia/dyspnea? Or was it a true co-infection? 

Excluding influenza, viral co-colonization is common in children, but actual co-infection with CAP is rare in adults [9]. Since her respiratory PCR was negative for *M. pneumoniae*, this suggests it was not colonizing oropharynx. Against the explanation that *M. pneumoniae* was throat commensal was the magnitude of her *M. pneumoniae* titers together with her highly elevated cold agglutinin titers, which was indicative of infection and her high IgM titers and cold agglutinin titers point to a primary pathogenic role [2,3].

Review of the literature on severe *M. pneumoniae* CAP reveals that although *Mycoplasma pneumoniae* is usually mild, clearly there are reports of severe CAP due to this organism in normal hosts [10,11,12,13,14,15,16,17,18]. It has been suggested that some strains of *M. pneumoniae* may elaborate a toxin which may be a virulence factor in severe cases with acute respiratory distress [19,20].

In the end, we were left with the original diagnostic dilemma regarding the etiology of her hypoxemia/dyspnea, i.e., if due to one or the other pathogen or the combined effect of both pathogens, i.e., EBV and *M. pneumoniae*. She slowly recovered on respiratory quinolone therapy, was discharged with less fatigue, and was no longer hypoxemic or dyspneic.

## 4. Conclusions

This case is instructive for clinicians in approaching patients with EBV IM and/or *M. pneumoniae* pneumonia and dyspnea.

## Figures and Tables

**Table 1:** EBV Infectious Mononucleosis: Radiographic Manifestations.

Adenopathy	Parenchymal Lung Abnormalities
**Common**	**Uncommon**
Bilateral hilar adenopathy (BHA)	Bilateral interstitial infiltrates ^a^
**Uncommon**	**Rare**
Mediastinal adenopathy	ARD Small pleural effusion ^b^ (unilateral/bilateral)

Note: ARDS = acute respiratory distress syndrome; ^a^ Usually associated with hilar/mediastinal adenopathy, but rarely may be present without BHA; ^b^ Usually associated with interstitial infiltrates, but rarely may be present alone. Adapted from Reference [9], Cunha, B.A.; Gian, J. Diagnostic Dilemma: Epstein–Barr virus (EBV) infectious mononucleosis with lung involvement or co-infection with Legionnaire’s disease? *Heart Lung* **2016**, *45*, 563–566.

**Table 2:** Differential Diagnosis of Elevated Cold Agglutinin Titers.

Infectious Causes	Non-Infectious Causes
Cold agglutinin titers usually high (>1:64)	
*Mycoplasma pneumoniae*	Cold agglutinin disease
Cold agglutinin titers usually low (>1:2–1:32)	
Respiratory pathogens:	SLE
Adenovirus	Multiple myeloma
Influenza	Waldenstrom’s macroglobulimemia
Non-respiratory pathogens:	Lymphoma
EBV	CLL
CMV	Sinus histocytosis
HCV	
Malaria	
Trypanosomiasis	
Coxsackie viruses	
Measles	
Mumps	
HIV	
SLE = systemic lupus erythematous EBV = Epstein–Barr virus CMV = cytomegalovirus CLL = chronic lymphocytic leukemia HCV = hepatitis C virus HIV = human immunodeficiency virus	

Note: Adapted from Reference [9], Cunha, B.A.; Gian, J. Diagnostic Dilemma: Epstein–Barr virus (EBV) infectious mononucleosis with lung involvement or co-infection with Legionnaire’s disease? *Heart Lung* **2016**, *45*, 563–566.
